# Clinical results of tricuspid valve replacement – a 21-case report

**DOI:** 10.1016/S1674-8301(10)60012-7

**Published:** 2010-01

**Authors:** Yu Zhuang, Jie Zhou, Mingdi Xiao, Zhongxiang Yuan, Chengbao Lu, Min Yu, Lei Lin

**Affiliations:** a Department of Cardiovascular Surgery, Shanghai Jiao Tong University Affiliated First People's Hospital, Shanghai, 200080,China.; b Department of English, College of Foreign Language, Guangxi University for Nationalities, Guangxi, 530006, China.

**Keywords:** tricuspid valve replacement, heart valve diseases, cardiac function

## Abstract

**Objective:**

To summarize the clinical experiences of 21 patients treated with tricuspid valve replacement (TVR) and investigate the surgical indications and methods.

**Methods:**

Data from 21 patients who underwent TVR from December 2002 to March 2009 were retrospectively collected and analyzed. The mean age was 48.86±15.37 years (range: 20-72 years). The underlying disease of the patients was classified as rheumatic (*n* = 10), congenital (*n* = 8), endocarditis (*n* = 2) or chest trauma (*n* = 1). Previous cardiac surgery had been performed in 12 patients (57.14%).

**Results:**

In-hospital death occurred in two patients (9.52%). Postoperative morbidities included cardiac failure (*n* = 2), bleeding related re-operation (*n* = 1), and plural effusion (*n* = 2).

**Conclusion:**

The early outcomes of TVR were acceptable. At the present time TVR can be performed through optimal perioperative management.

## INTRODUCTION

Tricuspid valve disease is a common lesion resulting from many other diseases and generally manifested as tricuspid regurgitation[1]. While mitral valve and/or aortic valve replacement is performed extensively, tricuspid valve replacement TVR is an uncommon procedure. The reason is that the tricuspid valve is amenable to repair, and the mortality of TVR is high[2]–[5]. For those with advanced severe tricuspid valve disease, the valve lesion is usually too complex to repair and medication is unable to attenuate symptoms. TVR might be the only choice for these patients. During the past decades, improvements in perioperative management have reduced the early mortality and morbidity after cardiac valve surgery[6]. However, there is still a high mortality associated with TVR[2]–[4]. In this study, we sought to evaluate the early results of TVR in a single medical center in China.

## MATERIALS AND METHODS

We retrospectively reviewed the data of 21 consecutive patients who underwent stand-alone TVR at our medical center between December 2002 and March 2009. During the study period, 1,293 valve replacements were performed, including the 21 TVRs (1.62%).

### Patient characteristics

The patient demographics are presented in ***Table 1***. The age of the patients ranged from 20 to 72 years (48.86± 15.37 years). The underlying diseases of the patients included rheumatic (*n* = 10), congenital (*n* = 8), endocarditis (*n* = 2) or chest trauma (*n* = 1). Previous cardiac surgery had been performed in 12 patients (57.14%). Nine patients had previous tricuspid valve repair (TVP) (42.86%). All the patients had tricuspid regurgitation (100%), and two of the rheumatic patients had tricuspid stenosis (9.52%).

**Table 1:** Patient characteristics

	*N*	Percent(%)
Gender (female)	15	71.4
		
NYHA class		
IV	5	23.8
III	10	47.6
II	6	28.6
Previous cardiac surgery		
MVR	6	28.6
BVR	2	9.5
TVP	1	4.8
VSD repair	1	4.8
Pacemaker implantation	2	9.5
Rhythm		
Atrial fibrillation	7	33.3
Sinus	11	52.4
Paced	2	9.5
LVEF≤40%	4	19.0
Ascites	7	9.5
Active infective endocarditis	2	4.8

LVEF: left ventricular ejection fraction; NYHA: New York Heart Association; MVR: mitral valve replacement; BVR: aortic and mitral valve replacement; TVP: tricuspid valve repair; VSD: ventricular septal defect

### Surgical technique

A midline sternotomy was performed in 15 patients, except in six in whom a right anterolateral thoracotomy was performed. Standard aortic and direct bicaval cannulations were performed in 15 patients. Femoral artery and venous cannulations were used in six patients. Eighteen TVRs were performed under mild hypothermia arrested heart cardiopulmonary bypass (CPB), two were performed under deep hypothermic circulatory arrest (DHCA), and one was performed under mild hypothermia beating heart CPB. Myocardial protection was achieved by antegrade cold-blood cardioplegia infusions.

A bioprosthesis was used in 11 cases and a mechanical valve in 10. The mean bypass time was 95.1±44.4 min (range: 50-205 min) and the mean cross-clamp time was 46.7±13.0min (range:25-78 min). Ultrafiltration was used in all the patients, and the mean volume of fluid removed was 2,113±765 ml. The average filtration volume per body weight was 36.1±6.6 ml/kg (range: 22.6-48.9 ml/kg).

### Statistical analysis

Data are presented as the proportions (%) and mean±standard deviation (SD) for categorical variables and numeric variables, respectively. Stata 10.0 was adopted for statistical analyses. The paired *t* test was used to determine differences in patient cardiothoracic ratio pre- and post-operation. A *P* value < 0.05 was considered significant for all tests.

## RESULTS

Two patients died from refractory cardiac failure (9.52%). They were both older patients (72 and 61 years old) with previous mitral valve replacement (MVR) and NYHA Class-IV. One patient was re-operated for bleeding. Two cases had right heart failure after surgery. One had previous aortic and mitral valve replacement (BVR), TVP, Class-IV and diabetes mellitus; the other had previous MVR, tricuspid valve repair, Class-IV and hypertension. In both cases the failure was successfully cured with dopamine, milrinone infusion and diuretics. A large volume of plural effusion occurred in two previous MVR, TVP, and Class-III patients was successfully cured with plural drainage, albumin and diuretics. The remaining patients received standard rehabilitation and were discharged with significantly improved mean cardiothoracic ratios (52.21%±8.84% *vs.* 73.95%±11.33%, *P* = 0.000) and cardiac function (I:7, II:10, III:2).

## DISCUSSION

The characterization of tricuspid regurgitation is the backflow of regurgitant blood into the right atrium during systole. There are often no apparent hemodynamic changes and clinical signs and symptoms among patients with mild or moderate tricuspid regurgitation because the right atrium is relatively compliant. However, when the regurgitation is severe, the right atrial and venous pressures rise and cause the signs and symptoms of congestive right heart failure, such as pulsatile jugular veins, painful hepatosplenomegaly, ascites, and peripheral edema. Symptoms may also arise from pulmonary hypertension, such as fatigue, weakness, shortness of breath, and exercise intolerance. The clinical manifestations of tricuspid stenosis are similar to those of tricuspid regurgitation, except that it has different initiating causes and pathophysiology. There is a persistent diastolic pressure gradient between the right atrium and right ventricle in tricuspid stenosis patients. Accordingly, tricuspid diseases sustained long enough may cause extensive compromise both locally (tricuspid valve itself and right ventricle) and systemically (especially the liver). Hence, any hemodynamically important tricuspid valve disease not amenable to medication should be considered for surgical intervention. Some surgeons are cautious in deciding to perform TVR because of its relatively high mortality (26%) and morbidities (59.5%)[2], especially for those patients with previous heart surgery. Singh et al[7] reported that TVP was superior to TVR. However, Moraca *et al*[4] reported that there's no evidence favoring TVP over TVR, and indicated that the operative mortality was similar in the two groups (18%±5% *vs.* 13%±4%, TVP *vs.* TVR, *P* = 0.64). According to our experiences, whether to repair or replace the valve depends on the tricuspid valve lesion. Repair may be suitable for mild or moderate lesions. As for those valves exhibiting severe degenerative diseases, diminished leaflet area, severe fusion of chordae or endocarditis, replacement may be a more reasonable choice. Furthermore, it is difficult to determine the optimal timing of the operation. Early surgery causes valve-related complications, but excessive delay may not reverse end organ damage, which results in many risk factors[2],[5],[8],[9], such as rheumatic etiology, reoperation, NYHA functional class III or IV, severe pulmonary hypertension, hepatic dysfunction, and ascites, all of which might contribute to poor follow-up outcomes. The two patients who died in our study group were re-operated rheumatoid patients, exhibiting large amounts of ascites and having NYHA functional class IV. Low cardiac output syndromes (LCOS) occurred after the operation, and hepatic failure occurred soon after LCOS. We believe that the operation should be undertaken before damage occurs to the end-organs, just after medication looses effectiveness. However, there are no standards and the timing of the surgery depends on the surgeon's experiences and skills.

In China, Xiao et al[9] reported a mortality of 13%, and Dong et al[10] reported a mortality of 4.3% following TVR surgery. The in-hospital mortality reported from abroad was higher (13-26%)[2]–[4], especially in those patients who were re-operated TVR (35.1%)[5]. We achieved a significantly lower mortality than this, and our data are comparable to other Chinese TVR mortality reports.

Patients undergoing TVR are typically high-risk with a high-percentage of reoperations, more complications, and end-stage heart functional class IV. Hence, myocardial protection might play an important role in these high risk patients. Considering the difficulty in separating the adhesions and injury of the enlarged heart through the original incision, right anterolateral thoracotomy was performed in six patients due to heart enlargement and long history of prior MVR or BVR in our group. In two cases, the right atrium was enormously enlarged and severe adhesions occurred in the superior and inferior vena cava. With femoral artery and venous cannulations and DHCA, we avoided the potential damage that could occur during peri-vena cava adhesion separation and decreased operation time. Tricuspid leaflets and the subvalvular apparatus were preserved as much as possible, so as to keep the continuity of the tricuspid valve and right ventricle, which contributes to heart function. In conditions of right ventricular dysfunction, excessive volume causes multiple organ edema and dysfunction, which worsen the progress of right ventricular dysfunction. Diuretics were routinely used to interrupt this vicious cycle. Ultrafiltration during the operation has also been reported to decrease myocardial edema and improve right ventricular function[11].

In summary, although it has a high post-operative mortality due to re-do operation, right heart failure and concomitant complications; the early operative outcome of TVR can be improved through improvements in myocardial protection during surgery, and choosing a suitable operation time.
